# Molecular Characterization of Bovine *Deltapapillomavirus* in Equine Sarcoids in Egypt

**DOI:** 10.1155/vmi/9773642

**Published:** 2025-01-03

**Authors:** Nader Maher Sobhy, Walid Refaai, Rahul Kumar, Christiana Rezk Bottros Youssef, Sagar Mal Goyal

**Affiliations:** ^1^Department of Animal Medicine, Faculty of Veterinary Medicine, Zagazig University, Zagazig, Sharkia 44511, Egypt; ^2^Veterinary Population Medicine Department and Veterinary Diagnostic Laboratory, College of Veterinary Medicine, University of Minnesota, St. Paul, Minnesota 55108, USA; ^3^Department of Surgery, Anesthesiology, and Radiology, Faculty of Veterinary Medicine, Zagazig University, Zagazig, Sharkia 44511, Egypt; ^4^Tennessee Department of Agriculture, C. E. Kord Animal Health Diagnostic Laboratory, Nashville, Tennessee 37220, USA; ^5^Microbiology and Immunology Department, Faculty of Pharmacy, Zagazig University, Zagazig, Sharkia 44519, Egypt

**Keywords:** Egypt, equine, papillomaviruses, sarcoid, whole genome sequencing

## Abstract

Bovine papillomaviruses (BPVs) commonly cause sarcoids in equines worldwide. Equine sarcoids (ESs) reduce the working ability of draft animals and produce untoward cosmetic changes in racing and dancing equine. In this study, nine horses and 16 donkeys with sarcoids were presented to Zagazig University Veterinary Clinic, Zagazig, Egypt. Of these, eight horses and six donkeys were found to be infected with BPV. On sequencing, all 14 viruses were found to be BPV1, which were distributed in two clades without specific differentiation among papillomaviruses (PVs) of donkeys, horses, and cattle. Comparison of 135 aa (319–454) of the sequenced L1 gene with reference strains revealed three conservative mutations (D346N, Q398E, and F441Y) and two nonconservative mutations (T348N and K351T). Illumina sequencing revealed that PVs of donkeys and horses were identical and had 98.5% identity with the closest reference sequence (KX907623) of BPV1. In addition, there was high identity among all genes except E5 and L2. The substitution ranged between 0.5% (nt) and 0.89% (aa) in E4 and 5.18% (nt) and 6.81% (aa) in E5. These results indicate that BPV1 is the main cause of ESs in Egypt without marked phylogenetic variation among PVs of cattle, horses, and donkeys.

## 1. Introduction

Sarcoids are locally invasive tumors of the skin and are the most common equine skin neoplasia [1]. The name “sarcoid” means “flesh-like” indicating the clinical appearance of the lesion [2]. Histologically, the mass of the proliferating cells consists of fibroblasts of the dermis. In some sarcoids, the epidermal layer is also thickened, although extensive ulceration interferes with the observation of this feature [3]. Equine sarcoids (ESs) occur mostly at the site of a previous skin injury. The growth can be solitary or in the form of multiple tumors. It can occur anywhere on the animal's body although the head, neck, limbs, and ventral abdomen are most commonly affected [4].

The ESs are classified into six visually recognizable types, e.g., occult, verrucose, nodular, fibroblastic, malignant, and mixed [5]. Occult sarcoids are identified as circular harsh alopecic areas with or without hyperkeratotic small cutaneous nodules. Verrucose sarcoids are harsher than occult ones with a more hyperkeratotic appearance and occupy wider areas. Nodular sarcoids are divided into types A and B of well-determined subcutaneous nodules with different sizes and numbers [6]. Fibroblastic types 1 and 2 are the most common annoying types that appear as raised, fleshy, ulcerated masses, which are more pedunculated in type 1 while broader and invasive in type 2 [5]. The mixed sarcoid has features of many types in the same tumor [6]. Malignant sarcoids appear as nodules or palpable tumors having lymphatic infiltration.

Bovine papillomavirus (BPV) is believed to be the cause of ES [1, 4]. The BPVs are a group of double-stranded DNA viruses, belonging to the genus *Deltapapillomavirus* in the *Papillomaviridae* family. The classification of PV types and subtypes is based on the similarity in the most conserved L1 sequence. Novel types, species, and genera should have > 90%, > 70%, and > 60% nucleotide identity, respectively [7]. Currently, BPV types are assigned into five genera: *Deltapapillomavirus* (BPV1, 2, 13, and 14), *Xipapillomavirus* (BPV3, 4, 6, 9, 10, 11, 12, 15, 17, 20, 23, 24, 26, 28, and 29), *Epsilonpapillomavirus* (BPV5, 8, and 25), *Dyoxipapillomavirus* (BPV7), and *Dyokappapapillomavirus* (BPV16, 18, and 22). In addition, BPV19, 21, and 27 belong to an unclassified genus [8]. A recent proposal suggested that classification should depend on E1, E2, and L1 and that the virus should be considered novel if it shares less than 90% identity in these genes [9]. The genus *Deltapapillomavirus* includes seven species, of which *Deltapapillomavirus* 4 (containing BPV1, 2, 13, and 14) is the most important for cattle. The genome consists of six early proteins (E6, E7, E1, E2, E4, and E5), two late proteins (L2 and L1), and a long control region (LCR) at the genome terminal [10]. The E2, E5, and E6 have significant oncogenic action stimulating sarcoid development with a minor oncogenic role of E7 [11].

The E5, E6, and E7 proteins of BPV1 increase matrix metalloproteinase 1 (MMP-1) promoter activity. MMP is a necessary proteinase for initiating the disintegration of the extracellular matrix and basement membrane to facilitate tumor cell invasion. The MMP-1 upregulation function is organized by activator protein-1 (AP-1)–binding site contributing to the invasiveness and spread of equine fibroblasts [12]. Two components of the AP-1 transcription factor complex (c-Jun and Fra-1) are activated and overexpressed by BPV1 in equine fibroblasts [13]. E2 protein was found to transactivate the promoter of MMP-9 via AP-1-binding sites [14].

Papillomaviruses (PVs) are usually species-specific. However, BPV1, 2, 13, and 14 are an exception because they can infect equines, ovines, and felines [15–17]. The exact method of sarcoid transmission is not fully understood. However, direct contact with BPV-infected cattle or horses or through flies carrying the virus is the most common mode of transmission [18]. Sarcoids may occur spontaneously or are related to a previous skin injury such as surgical manipulation, castration, punch biopsy, or laceration [4].

Two sets of primers (FAP59/FAP64 and MY09/MY11) are widely used to multiply L1 ORF for PV identification in humans and animals [19]. Almost all novel BPV types were discovered in bovines by using these primers. However, the BPV FAP59/FAP64 and MY09/MY11 primers are more suitable for detecting putative new BPV types. However, it cannot determine co-infection which needs more BPV type-specific primers [20]. Thus, both PCR primer systems are needed to identify co-infection. PCR using FAP59/FAP64 sets of primers was found capable of amplifying DNA from 87% (65/75) of the HPV types tested [19, 21]. Karlsen et al. recorded 91% sensitivity of MY09/MY11 and found it to be more sensitive [22].

Sarcoids can be treated by sharp excision, CO_2_ laser excision, cryotherapy, gamma radiotherapy, and electrochemotherapy. However, there is no evidence for preference for one treatment over the other [23]. Chemical therapy and/or surgery are indicated although immunological intervention may also have a role in eliminating PV infections [24].

ESs are often recurrent and may lead to the loss of valuable animals reared for racing and show [25]. A high recurrence rate was reported for ESs, which was significant among fibroblastic types [26]. Farmers in Egypt rely on donkeys and mules for draft and transport work. The lesions of sarcoids cause a reduction in the working ability of such animals and may also produce severe cosmetic damage to race and dancing horses. This study investigates the detection and characterization of PVs in clinical cases of ES in horses and donkeys in Egypt.

## 2. Materials and Methods

### 2.1. Ethical Approval

This study was approved by the Zagazig University-IACUC committee (ZU IACUC/2/F/332/2022).

### 2.2. Clinical Samples

Samples of skin lesions were collected from visually recognizable sarcoids from nine horses (*Equus caballus*) and 16 donkeys (*Equus africanus asinus*). These animals were brought to the Faculty of Veterinary Medicine Clinic, Zagazig University, Zagazig, Egypt, over a 2-year period. The animals were examined clinically for general health and parasitic infestation and sarcoids were tentatively assigned to various types based on their gross morphology [5] (Table 1).

### 2.3. Surgical Management

Animals were premedicated with xylazine HCL (0.5 mg/kg bw, I/V) and then deeply sedated with 10% chloral hydrate (6 gm/50 kg bw). Lesions were totally excised including 0.5 cm of the surrounding healthy tissue. The resultant wounds were either sutured and/or bandaged according to location, size, and amount of loose skin. A systemic course of an antibiotic (Borgal, 3 mL/50 kg·bw I/V daily/3 days) and an anti-inflammatory (Finadyne, 1 mL/45 kg·bw I/V daily/3 days) medication was administered along with a prophylactic dose of anti-tetanus serum (10 IU/kg·bw, S/C). Skin sutures were removed 10–14 days after surgery and the owners were contacted regularly for one year to check for the recurrence of sarcoids. After surgical removal, the sample was placed in phosphate-buffered saline (pH 7.4) and frozen at −20°C until DNA extraction.

### 2.4. Molecular Examination

DNA was extracted from these samples using a tissue DNA extraction kit (Vivantis). Two different sets of degenerate primers were used to amplify the conserved L1 gene. The primer sets (see Table 2) FAP59/FAP64 and MY09/MY11 amplify 478 and 450 base pairs (bp), respectively [21, 22]. PCR was performed in a 25 *μ*L reaction mixture using a Hot Star Taq master mix kit (Qiagen) in an automated thermocycler (Mastercycler, Eppendorf). The PCR conditions were 15 min at 95°C for initial denaturation followed by 35 cycles of 1 min at 94°C (denaturation), 1 min at 52°C (annealing), 1 min at 72°C (extension), and one final extension step of 10 min at 72°C. The PCR products were visualized in ethidium bromide–stained 1% agarose gel.

### 2.5. Sanger Sequencing and Sequence Analyses

All PCR products were purified using a PCR gel purification kit (Qiagen). Direct sequencing was performed at the University of Minnesota Genomics Center (UMGC) with the same forward and reverse primers. The quality of the sequence chromatograms was examined using “Sequencher 5.1” software. The sequences were aligned using the Clustal W option and phylogenetic analysis was performed in MEGA X software [27]. The sequences were submitted to GenBank with the following accession numbers, MT502094–MT502101 for *Deltapapillomavirus* 4 detected in horses and MT502102–MT502107 for those in donkeys.

### 2.6. Whole Genome Sequencing

Extracted DNA from lesions of horses and donkeys was combined in pool 1 and pool 2, respectively. Both pools were sent to UMGC for library preparation and Illumina MiSeq sequencing. Raw fastq files were trimmed by Trimmomatic (v 0.39) to remove adaptors. Host genome contamination was removed by bowtie2 (v 2.3.5). The sequence reads were assembled by the *de novo* option in CLC Genomic Workbench 6.0.1. Analysis of the extracted contigs and ORF prediction was performed by the BLAST (tBlastx) option in NCBI and the ORF finder tool (https://www.ncbi.nlm.nih.gov/orffinder), respectively. The genome was illustrated by the IBS tool (https://ibs.biocuckoo.org/citation.php). Protein GRAVY was calculated by using the Kyte and Doolittle method on sequence manipulation suite (https://sites.ualberta.ca/%7Estothard/javascript/protein_gravy.html).

## 3. Results

### 3.1. Clinical Examination

Sarcoids in 14 of 25 animals (8 horses and 6 donkeys) were confirmed to be caused by PV. Most confirmed cases were in males over 5 years of age (Table 1). All PV-infected animals were also found infested with parasites such as *Strongylus*, *Oxyuris*, and *Ascaris* (Table 1). Common sites for lesions were distal fore and hindlimbs below the carpus and hock (data not shown). In some animals, the lesions appeared in more than one location. As many as 31% of the animals showed recurrence of lesions within a year of surgical removal.

### 3.2. Gross Lesions

**Figure 1:**
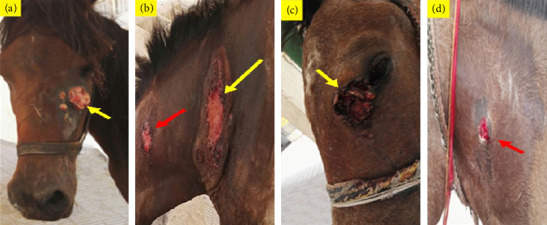
Sarcoid, skin, horse. (a) Fibroblastic sarcoid on face. Note irregular multinodular mass below the eye; (b) malignant sarcoid (yellow arrow) on the caudolateral aspect of the neck and small occult ulcerated sarcoid (red arrow) on the middle aspect of the neck; (c) mixed sarcoid (verrucous–fibroblastic) adjacent to the medial canthus of the eye; (d) occult (ulcerated) sarcoid on the left side of the neck.

**Figure 2:**
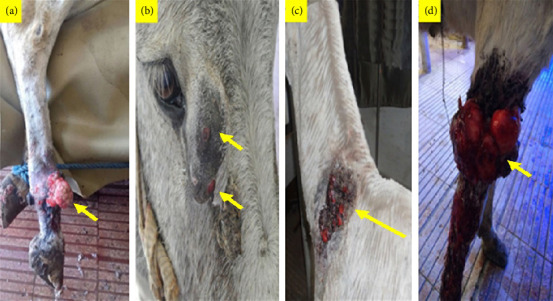
Sarcoid, skin, donkey. (a) Fibroblastic sarcoid on the dorsolateral aspect of the fetlock joint; (b) mixed sarcoid (verrucous–occult) below the eye; (c) mixed sarcoid (verrucous–fibroblastic) on the lateral aspect of the neck; (d) fibroblastic sarcoid on the dorsolateral aspect of the stifle joint.

Infection with PV was confirmed mostly in verrucose, fibroblastic, malignant, and mixed lesions (Table 1 and Figures 1 and 2). Briefly, a large mass with an ulcerated hemorrhagic surface resembling exuberant granulation tissue was tentatively diagnosed as a fibroblastic sarcoid (Figures 1(a), 2(a), and 2(d)). Palpable, visible, and invasive lesions that spread widely and extended subcutaneously were identified as malignant sarcoids (Figure 1(b)). Occult sarcoids showed focal areas of alopecia, hyperkeratosis, and hyperpigmentation with an ulcerated surface (Figure 1(d)). A wart-like growth with a peripheral dry, rough surface was classified as a verrucous type. Mixed sarcoid (verrucous–fibroblastic) had multiple wart-like masses surrounded by and mixed with hemorrhagic, ulcerated masses resembling granulation tissue (Figures 1(c) and 2(c)). Small wart-like growths surrounded by large hyperkeratotic and hyperpigmented areas below the eye were also mixed type (verrucous–occult) (Figure 2(b)).

### 3.3. Molecular Detection and Phylogenetic Analysis

**Figure 3:**
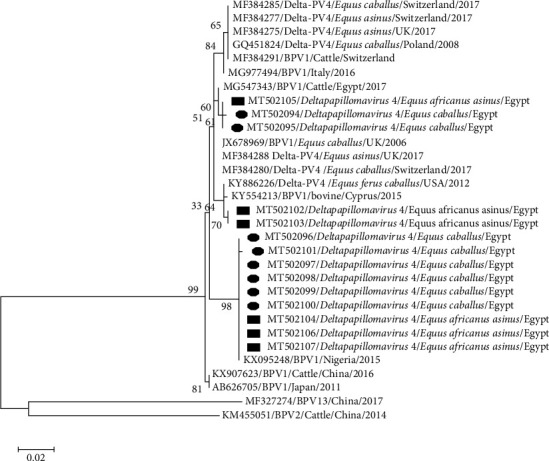
Phylogenetic analysis of the L1 gene of papillomaviruses from sarcoid lesions of donkeys and horses using the best-fit maximum likelihood model (Tamura 3-parameter + G) based on the lowest BIC score (Bayesian Information Criterion).

PCR with the MY09/MY11 primer set confirmed the presence of PV in eight horses and six donkeys while FAP59/FAP64 primer set revealed no bands. Sanger sequencing of all 14 sarcoids revealed the presence of BPV1 with a common ancestor, which was separated from BPV2 and BPV13. They were distributed in two clades without any specific differentiation among donkey, horse, and cattle PVs. The first clade was found in five samples (two horses and three donkeys), with 98%–100% nucleotides (nt) identity with each other and 99%–100% nt identity with the newest Egyptian cattle isolate (MG547343). This clade was similar to the European strains of BPV1 in ES. The second clade of nine samples (3 donkeys and 6 horses) had a maximum nt identity (100%) with Nigeria bovine isolate (KX095248) (Figure 3). Identity among horse and donkey PVs was 97%–100% while identity with reference equine and bovine strains was 97%–98%.

A comparison of 135 amino acids (aa) (319–454) of the sequenced L1 protein in all 14 samples with reference strains revealed five mutations. Three mutations were conservative (D346N, Q398E, and F441Y) where the replaced aa had the same size, biochemical properties, hydrophobicity, and charge. Nonconservative mutation was observed between threonine (T) and asparagine (N) at 348^th^ aa and between positively charged lysine (K) and uncharged T at 351^st^ aa position. Further substitution analysis revealed that D346N was present in three animals (1 donkey and 2 horses) with malignant sarcoid type; Q398E and K351T were observed in two donkeys with multi-sarcoid lesions; F441Y was detected in 3 donkeys and 6 horses and matched with a Nigerian isolate from cattle (KX095248.1) (Supporting Figure S1).

### 3.4. Complete Genome Sequencing

**Figure 4:**
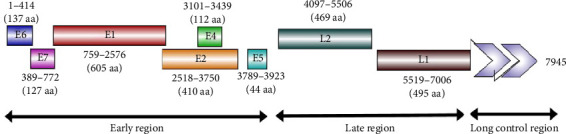
Arrangement of BPV1/Egypt/2019/MT459820 genome.

The whole genome sequences obtained from pools of donkey and horse samples were identical, with 98.5% nt identity with the nearest reference sequence of BPV1 (KX907623.1). The genome consisted of 7945 nt that encoded 2399 aa. Eight ORFs were identified for six early proteins (E6, E7, E1, E2, E4, and E5) and two late proteins (L2 and L1) plus the LCR (Figure 4). The sequences from donkeys and horses were submitted to GenBank as a single sequence with accession number MT459820.

Two zinc-binding domains (ZBDs), CxxC (29/30) CxxC, with 36 aa in between were observed in the E6 protein, while only one ZBD was detected in the E7 protein with a lack of retinoblastoma tumor suppressor-binding domain (LxCxE). The expected ATP-binding site (GPPNTGKS) was detected at 432–440 aa of E1. E4 ORF started with methionine and its protein completely overlapped the E2 ORF. A rich proline content (15 proline residues out of 112 aa) was detected in E4. The common leucine (L)-rich region that contains a hydrophobic transmembrane domain was present in E5.

The LCR consisted of 939 bp and extended from the end of L1 (7096) to the start of E6. Nine E2 binding sites (E2BS, ACCN6GGT) were identified at 7112–7124, 7274–7286, 7317–7329, 7419–7431, 7500–7512, 7529–7541, 7669–7681, 7690–7702, and 7805–7817. Extra putative modified E2BS (ACCN2GGT) was identified at 7932–7940. Only one E1 binding site (E1BS) (ATTGTTN3AACAAT) was detected at 7850–7865. Two polyadenylation sites (AATAAA) were detected at 5 terminal parts of LCR at 7065–7071 and 7001–7007 upstream in the terminal part of L1. Two TATA boxes were identified at nt 7791 and 7913 of the URR 3 terminal part.

**Figure 5:**
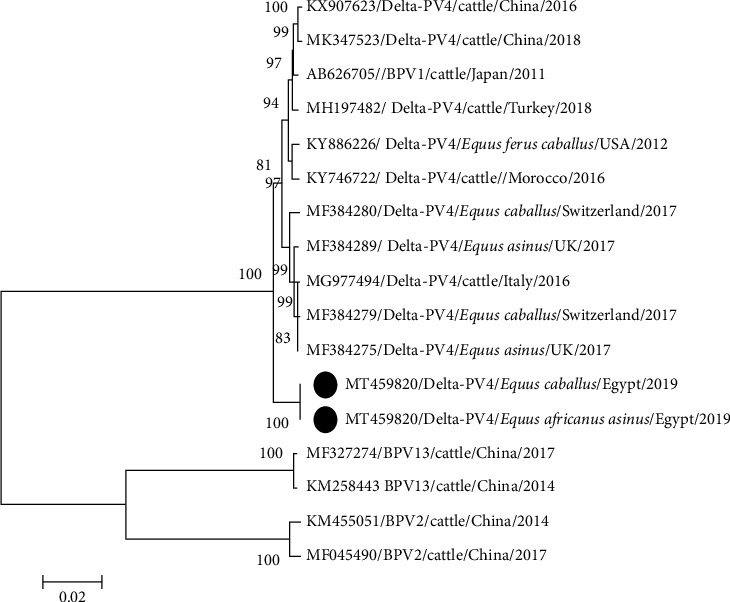
Phylogenetic analysis of the complete genome of two papillomaviruses obtained from sarcoid lesions of donkeys and horses in the best-fit maximum likelihood model (Tamura 3-parameter + G) based on the lowest BIC score.

Phylogenetic analysis of the whole genome indicated that donkey and horse PVs (MT459820) were identical. The sequence clustered with BPV1 strains but in a separate clade of the constructed phylogenetic tree and appeared as an ancestor of European and Asian strains (Figure 5). Comparison with the closest reference strain (KX907623.1) revealed high identity (99%) among all genes except E5 and L2 which had 95% and 98% nt identity and 93% and 99% aa identity, respectively. The substitution ranged between 0.5% (nt) and 0.89% (aa) in E4 and between 5.18% (nt) and 6.81% (aa) in E5 (Table 3). Change in E5 gene proteins also changed GRAVY values from 1.627 in reference strain (KX907623) to 1.511.

## 4. Discussion

Sarcoids are common in tropical and subtropical countries like Egypt. New types of sarcoids have been discovered in different animal species with advances in diagnosis and genome sequencing technology [28]. Most sarcoid cases in this study were received in summer and autumn. This may be attributed to warm weather stress and related immunosuppression and/or abundance of vector flies that may play a role in virus transmission [29]. Most lesions were detected in male animals, which are more susceptible to abrasions and viral infection due to hard fieldwork or routine fighting. Most cases had lesions on limbs as reported in previous studies [30, 31]. Multiple lesions in the limbs, below the eyes, and the neck may be attributed to nibbling behavior due to irritation or direct contact with a common fomite used for scratching. The presence of multiple lesions is often considered an indicator of infectious causes of sarcoid tumors [4].

All animals in this study were positive for parasitic infestation. This may lead to immunosuppression, which may increase the risk of tumor development by providing an opportunity for the insertion of active oncogenes in the host genome [32]. This may also interfere with spontaneous recovery from the tumor [24].

Recurrence after surgical intervention was reported by some owners, which may be attributed to the presence of BPV in the subjacent tissue around the surgical margins [33]. The use of surgically removed sarcoids for nucleic acid extraction may have increased the overall diagnostic sensitivity by PCR than that obtained with superficial swabbing or scrapings [34].

Phylogenetic analysis revealed clustering of PV strains of equine (horses and donkeys) and bovine origin irrespective of their continent or country of isolation. Koch et al. [35] reported similar findings based on E5 and LCR analysis. Clustering samples in two clades indicates the presence of two different substrains in the same locality. Geographical segregation was detected only among sequences of samples from different continents (Africa, South America, Australia, and Europe) depending on sequence variants in LCR [36]. Analysis of L1 sequences revealed that Asian strains (Chinese and Japanese) appeared as ancestors to the more recent African and European strains. In agreement with Peng et al. [37], the Asian strains were ancestors of European strains [37]. However, the phylogenetic analysis of the complete genome length shows that Egyptian strains were ancestors of both Asian and European strains (Figure 5). The results emphasize the hypothesis of Trewby et al. [36] that Africa is a possible origin for the European strains of BPV1. However, an examination of additional samples from Africa is required for confirmation. Because of the limited availability of BPV1 sequences in GenBank from Africa, we could not include many African strains in our phylogenetic analysis.

In general, the conservative mutation in late proteins often has a milder effect on protein function than the nonconservative ones, often with deleterious effects [38]. The K351T mutation may affect virus-cell attachment, leading to increased numbers of free viruses causing multiple sarcoids in donkeys. Knappe et al. [39] correlated the K mutation in L1 to a decrease in binding affinity to cell receptor molecules. The presence of D346N mutation in PVs of donkeys and horses with malignant sarcoid type and not in other types points to the possible role of this point mutation in diffusion of virus in the affected area. Absence of F441Y mutation in BPV1, 2, and 13 of all affected animal species from different countries except the Nigerian isolate of cattle (KX095248.1) may be specific to African isolates. More in vivo and in vitro investigations are needed to confirm the significance of mutations in L1 protein.

The annotated complete sequences obtained from ES had the same order of early and late proteins plus LCR [37]. There was no significant variation in the length of the individual ORFs [40]. The absence of retinoblastoma protein in E7 is associated with overexpression, progression, and tumor metastasis [41]. A proline-rich region in E4 is also found in human PVs of other genera and has a similar mechanism of regulation [42]. The L-rich region in E5 is critical for functional dimer assembly and cell–cell fusion and is commonly found in BPV1, 2, and 13 [43]. Most BPV1 have 10–12 E2BS, and the identified sequence has nine E2BS with identical ACCN6GGT motifs, while the tenth E2BS missed four internal nt to the flanking motifs that are necessary to produce an enhanced E2 response. The internal sequences of the flanking motifs do not participate in any dramatic effect to enhance activity [44]. We detected one site of E1BS, which can be absent in the LCR of some BPV1 [37]. The crucial TATA box, which is necessary for viral transcription, was also detected. Mutation in E5 was mainly within the second half of the gene, which agrees with Chambers et al. [2] and indicates that this region may be important in disease pathogenesis. Decreased GRAVY and changes in E5 hydrophobicity may also alter its functions.

## 5. Conclusions

Molecular methods diagnosed PVs causing sarcoids in horses and donkeys. Because of the small number of samples, it is not possible to confirm that BPV1 is the most prevalent type in Northern Egypt, a major location for Arabian horses. However, BPV1 is regarded as the main viral cause of ES, as supported by previous studies. No marked differentiation was observed among BPV1 of horses, donkeys, and cattle. The significance of variants between sequences needs further investigation.

## Figures and Tables

**Table 1:** Data on horses and donkeys showing sarcoids caused by PVs.

	Horses	Donkeys
Sarcoid type: confirmed (suspected)		
Occult	0 (1)	0 (0)
Verrucose (warty)	2 (2)	2 (4)
Nodular	1 (2)	0 (2)
Fibroblastic	2 (2)	2 (5)
Malignant	2 (2)	1 (1)
Mixed	1 (1)	1 (4)
Sex		
Male	7	5
Female	1	1
Age		
< 5	3	5
> 5	5	1
The season when lesions first appeared		
Summer	5	4
Autumn	2	2
Winter	1	0
Associated problem		
*Strongylus* spp.	5	4
*Oxyuris*	2	1
*Ascaris*	1	1

**Table 2:** The primers that used for detection of papillomavirus in equine sarcoids.

FAP59/FAP64	5′‐TAACWGTIGGICAYCCWTATT‐3′ 5′-CCWATATCWVHCCATITCICCATC‐3′

MY09/MY11	5′‐GCMCAGGGWCATAAYAATGG-3′ 5′‐CGTCCMARRGGAWACTGATC‐3′

**Table 3:** Nucleotide and amino acid substitutions in BPV1/Egypt/2019/MT459820 genome and identity in comparison with the closest strain BPV1/isolate SY-12 (KX907623.1).

Gene	Nucleotides				Amino acids
	I	SN (%)	I	SA (%)	Position in translated ORF
E6	99	5/414 (1.2)	99	2/137 (1.45)	R (38), Q (99)
E7	99	4/384 (1.04)	100	0/127 (0)	0
E1	99	20/1818 (1.1)	99	5/605 (0.82)	L (54), A (109), S (117), A (124), G (137)
E2	99	11/1233 (0.89)	99	4/410 (0.97)	A (104), V (168), P (259), D (316)
E4	99	2/339 (0.5)	99	1/112 (0.89)	K (101)
E5	95	7/135 (5.18)	93	3/44 (6.81)	M (24), S (40), N (41)
L2	98	28/1410 (1.98)	99	6/469 (1.27)	A (93), I (289), K (303), P (379), P (415), T (433)
L1	99	22/1488 (1.47)	99	5/495 (1.01)	N (31), D (55), P (176), S (268), V (441)
LCR	99	9	—		Deletion in one nt (87)
Total	99	99/7095 (1.39)	—	26/2399 (1.08)	—

*Note:* SN, No. of substituted nt/total nt; SA, No. of substituted aa/total aa; I, percentage of identity.

## Data Availability

The data used and/or analyzed in this study are available from the corresponding author upon request.

## Supporting Information

Figure S1: the point mutation along the sequenced part of the L1 protein.
