# Supplementary figures and images for: Molecular Characterization of Bovine Deltapapillomavirus in Equine Sarcoids in Egypt
Source: Vet Med Int. 2025 Jan 3;2025:9773642. doi: 10.1155/vmi/9773642 (PMC11724032; doi:10.1155/vmi/9773642)

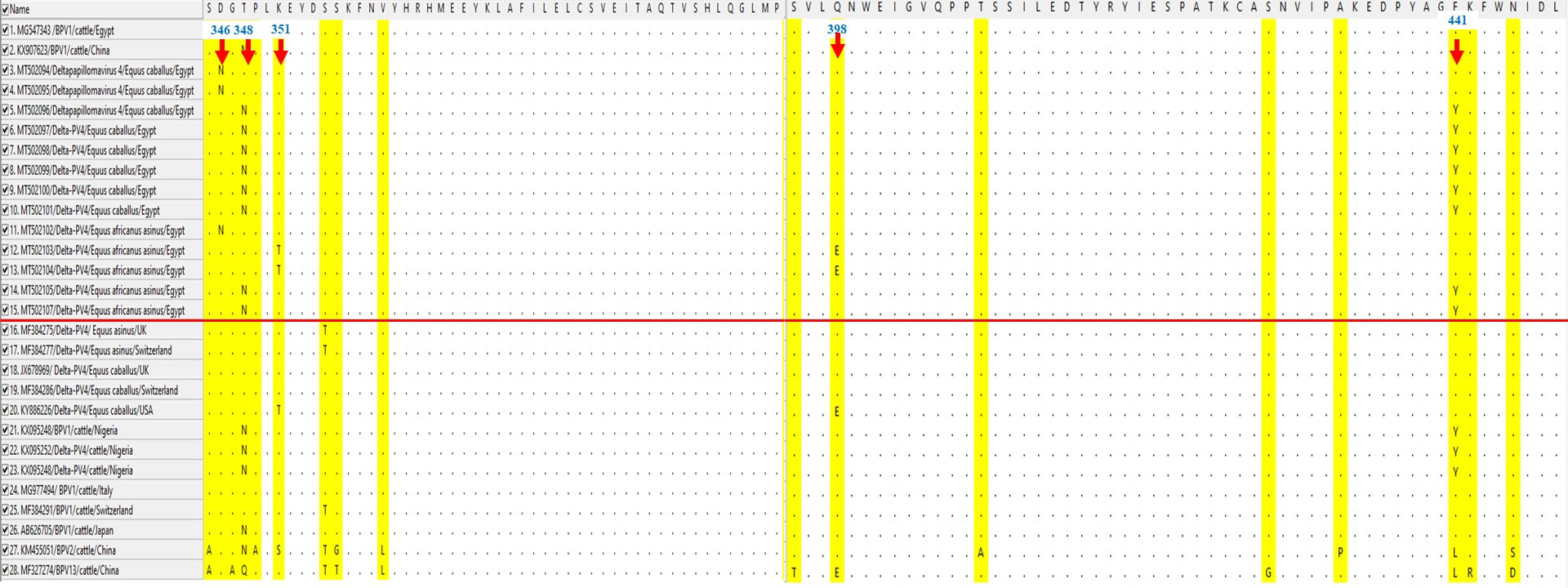

Supplement: Supporting Information — Additional supporting information can be found online in the Supporting Information section. [file 9773642.f1.jpg]
